# Chronic Bee Paralysis Virus in Honeybee Queens: Evaluating Susceptibility and Infection Routes 

**DOI:** 10.3390/v6031188

**Published:** 2014-03-11

**Authors:** Esmaeil Amiri, Marina Meixner, Ralph Büchler, Per Kryger

**Affiliations:** 1 Department of Agroecology, Aarhus University, 4200 Slagelse, Denmark; E-Mail: e.amiri79@gmail.com; 2 LLH Bieneninstitut Kirchhain, Erlenstr. 9, 35274 Kirchhain, Germany; E-Mails: Marina.Meixner@llh.hessen.de (M.M.); Ralph.Buechler@llh.hessen.de (R.B.)

**Keywords:** honeybee, chronic bee paralysis virus, queen susceptibility, worker to queen transmission, throphallaxis

## Abstract

Chronic bee paralysis virus (CBPV) is known as a disease of worker honey bees. To investigate pathogenesis of the CBPV on the queen, the sole reproductive individual in a colony, we conducted experiments regarding the susceptibility of queens to CBPV. Results from susceptibility experiment showed a similar disease progress in the queens compared to worker bees after infection. Infected queens exhibit symptoms by Day 6 post infection and virus levels reach 10^11^ copies per head. In a transmission experiment we showed that social interactions may affect the disease progression. Queens with forced contact to symptomatic worker bees acquired an overt infection with up to 10^11^ virus copies per head in six days. In contrast, queens in contact with symptomatic worker bees, but with a chance to receive food from healthy bees outside the cage appeared healthy. The virus loads did not exceed 10^7^ in the majority of these queens after nine days. Symptomatic worker bees may transmit sufficient active CBPV particles to the queen through trophallaxis, to cause an overt infection.

## 1. Introduction

The honey bee (*Apis mellifera*) is host of more than a dozen viruses [1,2,3]. One of the first viruses isolated and described in honey bees is Chronic Bee Paralysis Virus (CBPV) [4,5], causing chronic paralysis in adult worker bees [4,5,6].

The virus has a worldwide distribution and can be detected throughout the year. However, the infection often remains covert with asymptomatic bees [4,7]. Outbreaks of severe disease symptoms are erratic, but appear more frequently in spring and summer at the peak of colony development [7]. Infected bees are recognized by characteristic clinical symptoms (reviewed in [8]): Type 1 infection is associated with abnormal trembling of the wings and body, accompanied by bloated abdomen, clustering of sick bees in front of the hive entrance or bees crawling on the ground. Typical for type 2 is the loss of almost all hair on the abdomen, leading to a dark, shiny and greasy appearance. In the beginning, bees with symptoms of Type 2 are still able to fly, but after a few days they become flightless and die [8]. An outbreak of CBPV can lead to a severe loss of worker bees and to the collapse of the colony [9]. 

In symptomatic bees, CBPV has been found at high levels in the brain [6,10,11]. In Situ Hybridization showed that especially somata and neuropile regions of paralyzed bees are affected [11]. In particular, CBPV was observed in higher neuronal regions, like the optical and sensorial neuropiles of the brain; including mushroom bodies and central complex [11]. 

Two main routes of CBPV transmission are known: contact between adult bees when healthy bees are crowded together with infected individuals [12], and spread of infectious particles in the feces of paralyzed bees that are taken up orally by healthy nestmates [13]. Crowded conditions favoring these two routes occur frequently in growing bee colonies during the spring and early summer. In experimental studies, bees can be successfully infected by feeding the virus mixed into sugar solution, by topical application of viral particles, or by direct injection [4,14,15]. Experimentally infected adult bees show symptoms after about 5–6 days, similar to those of naturally infected bees [15,16,17]. A higher number of virus particles is needed to infect bees by topical application and by oral infection, as compared to injection [4,12,16]. A recent study showed that CBPV replication was more effective in individual bees inoculated *per os* compared to bees inoculated via the cuticle [18]. 

Several viruses of honey bees are known to be associated with, and vectored by, parasites. Black Queen Cell Virus (BQCV) has been linked to an infection with the gut parasite *Nosema spp* [19,20]. The invasive parasitic mite, *Varroa destructor*, is a known vector of both Deformed Wing Virus (DWV) [21,22] and Acute Bee Paralysis Virus (ABPV) [23]. Experimental evidence suggests that *Nosema ceranae* and CBPV may act synergistically to kill caged bees [18], however, evidence from a field study remains ambiguous [24]. Early studies did not find any contribution of varroa mites to the dissemination of CBPV [20,24,25], but in a later study up to 10^4^ virus particles were identified in individual mites [26]. The same study implicated ants (*Formica rufa* and *Camponotus vagus*) as a potential secondary host and reservoir of CBPV around apiaries [26], although their role in spreading the infection remains unclear. 

Using real-time PCR, CBPV can be detected in all developmental stages of the bee from egg to adult [6], but only adult bees show of the disease [8]. Virus presence has been detected in all castes (Worker, Queen, and Drone) [6,20,27,28]. Guard bees showed a significantly higher virus load than forager and hive bees, and also as drones [6]. Often, the queen and a few workers are the only remaining individuals in colonies collapsing due to CBPV [8]. From this observation, we develop the hypothesis that mechanisms exist to protect the queen against CBPV infection, when compared to the rest of the individuals in the colony. The social context of a honey bee colony depends on the presence of the queen, who maintains colony coherence via her pheromones. The queen is continuously attended to and fed by so-called retinue workers attracted by the pheromones [29]. Thus, behavioral patterns may exist that protect the queen from infection by symptomatic bees and allowing her to remain relatively unaffected [30]. Alternatively, queens may be less susceptible to CBPV compared to workers. However, we have often found early losses of young queens to be associated with a CBPV infection. We here present data to test both hypotheses. We first show that queens are equally susceptible to CBPV as workers. Secondly, we compare different ways of virus transmission, demonstrating the possible role of the queen’s interactions with her surrounding workers.

## 2. Results and Discussion

Two separate experiments were performed to test the susceptibility to CBPV and the possible transmission pathway of CBPV between workers and queen. 

### 2.1. Testing Susceptibility (Experiment 1)

#### 2.1.1. Observation of Symptoms and Mortality

**Figure 1:**
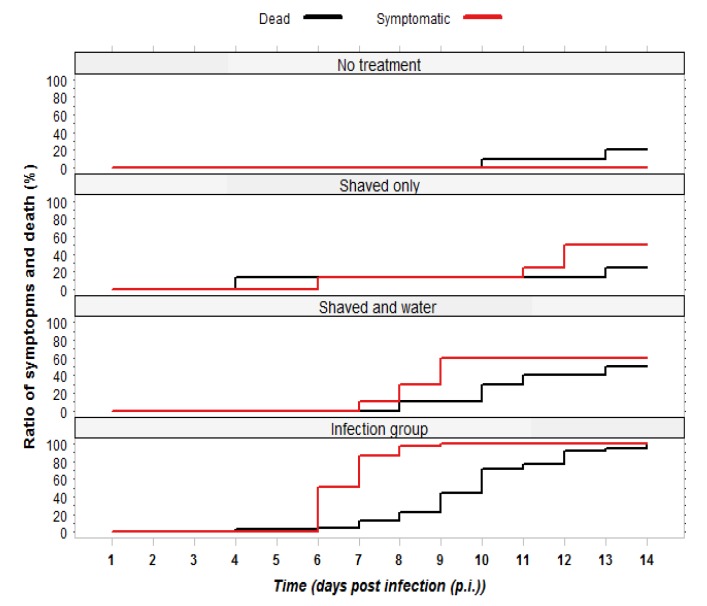
Development of Chronic Bee Paralysis Virus (CBPV) symptoms and cumulative mortality in queens of four experimental groups. The development of symptoms was faster in the infection group, which had a mortality rate of 100%.

In the first experiment, we investigated whether queens have a different susceptibility to CBPV than that described for worker bees in the colony. Queens (n = 39) that were infected as described in Experimental Section 3.2.1 started showing symptoms from day 6 p.i. and by day 9 all of them were symptomatic. Mortality in this group set in on day 6 and reached 100% by day 14 p.i. (see Figure 1). The most frequently observed symptoms were trembling of legs, and spread and disjunct wings. Some of the symptomatic queens had bloated abdomens: after dissection, their body cavity was found full of haemolymph and the honey sac was dilated. The progression of the infection followed a similar pattern as that described for workers [12,13], with the first symptoms appearing after 5–6 days p.i., and mortality of 100%. 

Symptoms were also observed in the two control groups shaved+water and shaved only, starting on day 7 p.i., see methods for details. Shaving is the accepted method for infection of bees with CBPV [8,12], as it allows the virus to enter the haemolymph. Still, the progression of symptoms was much slower and not all queens became symptomatic (see Figure 1). In the shaved+water control group, six queens developed symptoms, three of which died by day 14. Two more queens died without developing symptoms, but later were found to harbor moderate virus levels. In the shaved only group, four queens developed symptoms, one of which died. Another queen in this group died already on day four and was later found free of CBPV (Figure 1). In the third control group without any treatment, one queen died on day 10 and one on day 13 p.i. (see Figure 1). Since we sacrificed all remaining queens on day 15, we are unable to deduce the level of mortality in the control groups. 

#### 2.1.2. RT-PCR Confirmation of Infection Status

Within the infection group, 34 of 39 queens showed high virus loads above 10^7^ copies (7.0 × 10^8^–2.3 × 10^11^ copies per queen head), while 5 queens showed a moderate CBPV load of 10^4^–10^6^ (range 1.5 × 10^4^–2.4 × 10^6^). Some queens were also found to be infected with a low level of DWV (range 2 × 10^2^–4.9 × 10^6^), but we did not detect viruses of the ABPV complex in any of the queens. Out of 28 queens in the three control groups, symptoms and mortality were observed in 11 and 9 individuals, respectively. However, the virus loads of the 11 symptomatic queens remained comparatively low, ranging from 1.7 ×10^4^ to 2.7 ×10^7^. An analysis of variance based on rank transformed data showed that the virus loads between the infection group and the three control groups were significantly different (Table 1). 

**Table 1:** Analysis of variance between infected and three control groups of queens.

Source of variation	Degrees of freedom	Sum of squares	Mean of squares	F value	Pr (>F)
Groups of queens	3	14,294	4764.8	28.18	0.000
Residual	63	10,653	169.1		

**Figure 2:**
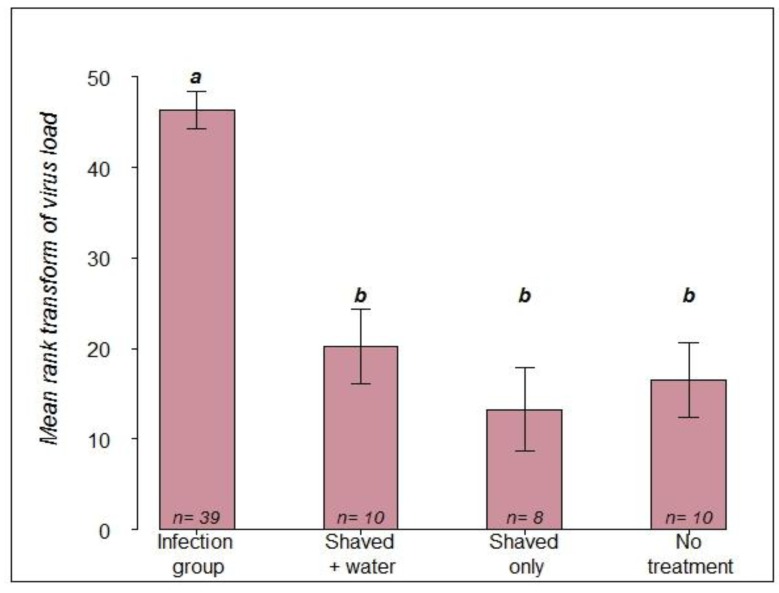
Comparison of mean rank transformed viral load among the infection group and the three control groups based on the LSD test. Means with common letters are not significantly different *p* ≤ 05.

Furthermore, a comparison of the mean values by a LSD test showed no significant differences between the three control groups (Figure 2). The virus load did not differ significantly between control queens dying during the experiment and control queens that survived until termination of the experiment on day 15 (*p*-value > 0.58). 

The results of this experiment clearly show that there is no difference between queens and workers in susceptibility to CBPV. The post mortem virus titers in the majority of the infected queens reached levels from 10^8^ to above 10^11^ copies per head, and thus fell in the range of virus levels published for symptomatic workers [6,17]. Although all queens of the infection group were treated on the same day with the same virus suspension, five individuals only reached a moderate level between 10^4^ and 10^6^ copies per head. It is possible that not the entire volume of virus suspension was absorbed, or the shaving of the thorax was not effective enough to open the queens’ cuticle. The lower viral titer in these five queens may thus be the result of a low level virus infection and viral replication within the queen, rather than a reduced susceptibility. In spite of these lower CBPV titers all five queens developed CBPV symptoms and died. We can exclude DWV and ABPV complex infections as cause of death, due to our testing of infection with RT-qPCR.

All queens in this experiment were taken out of production hives and approximately one year old. Thus, they already may have carried an unapparent infection before the experiment started, as this possibility is known from previous published reports [20,27,28]. Shaving the thorax, which is considered to open the cuticle, may allow virus particles already present on the surface to gain access to the interior of the queen. The results from control group 1 (shaved+water) also indicate that exposure to liquid may facilitate this process. It may also be possible that stress induced by the experimental situation in the laboratory initiated virus replication in a previously covert infection [30]. We cannot exclude the possibility of airborne virus transmission in the incubator, but thus far, no reports of airborne transmission of CBPV are known [8].

### 2.2. Comparing Infection Routes (Experiment 2)

#### 2.2.1. Observation of Symptoms and Mortality

In the second experiment, three contact options between workers and queens were compared to evaluate possible transmission routes of CBPV between worker bees and the queen in the colony. In group A (queens kept in cages together with symptomatic worker bees in the incubator) 23 of 29 individuals started showing symptoms 5–6 days p.i.. Similarly, 15 of 32 queens in group B (topical virus application, queens kept with healthy workers in cage in the incubator) showed symptoms by day 6. In contrast, only three of the 29 queens in group C (queens kept together with symptomatic bees in shipping cages inside mating nucs) started to show symptoms by day 9 after introduction. However, recognition of symptoms inside the queen shipping cages was more difficult and early symptoms may have been missed.

#### 2.2.2. RT-PCR Confirmation of Infection Status

The result of the virus quantification showed a clear differentiation pattern among the three experimental groups of queens. Most queens caged with symptomatic bees in the incubator (group A) had high viral loads. Twenty-five of 29 queens showed a high range between 3.0 × 10^9^–4.4 × 10^11^ virus copies, while the four remaining queens showed a moderate level (4.4 × 10^4^–1.4 × 10^6^ virus copies per head). Among these four, three died even before onset of symptoms. From 32 queens with a topical application of CBPV (Group B), 15 queens developed symptoms and had high virus level (2.9 × 10^9^–3.6 × 10^11^), but for 17 individuals no symptoms were observed and they showed no virus or only a moderate virus load (1.4 × 10^4^–1.5 × 10^7^). Among these, four queens died before onset of symptoms, maybe for reasons not related to viral infection, such as handling or experimental stress. Comparatively low viral titers were quantified in the majority of queens kept with symptomatic workers in queen shipping cages inside mating nucs (Group C). Only six of these queens had more than 10^8^ viral copies per individual, and among these four died before termination of the experiment without symptoms having been noticed. The remaining 23 queens had fewer than 10^7^ copies per queen. Analysis of variance from the rank transformed viral loads resulted in significant differences among the three experimental groups A, B and C (Table 2).

**Table 2:** Analysis of variance comparing rank transformed viral loads among groups of queens infected applying three different experimental setups.

Source of Variation	Degrees of freedom	Sum of squares	Mean of squares	F value	Pr (>F)
Groups of queens	2	17,215	8607.6	17.2	0.000
Residual	87	43,527	500.3		

**Figure 3:**
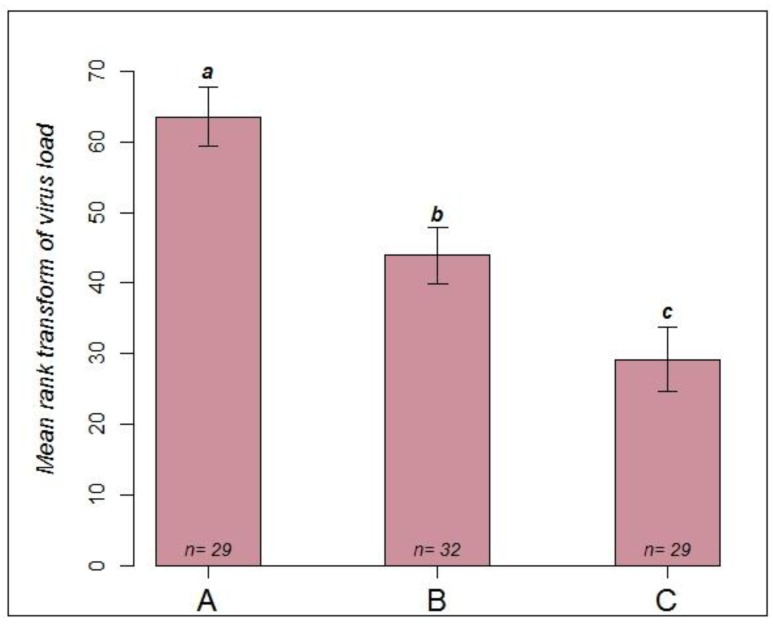
Comparison of mean rank transformed viral load among three groups of queens infected by different routes: Group **A**: queen with symptomatic bees in cage, in incubator, terminated on day 6 p.i.. Group **B**: topical infection with virus suspension, queen with asymptomatic bees in cage in incubator, terminated on day 6 p.i.. Group **C**: queen with symptomatic bees in shipping cage, in mating nuc with healthy bees, terminated on day 9 p.i..

A LSD test was used to compare the mean rank transformed viral load among these three groups. Significant differences were observed among all pairs of groups (Figure 3). Paralysis symptoms in the 15 successfully inoculated queens of group B were obvious after 5–6 days p.i.. Quantitative RT-PCR measured titers above 10^9^ virus copies in the majority of these queens. This is similar to the result we obtained in experiment 1 and also to virus levels found in workers after experimental inoculation [6]. However, virus transmission apparently was less successful in 17 queens, and they survived without symptoms for the 6 days of the experiment. It thus seems that virus titer above 10^7^ copies in the head is a good indicator of disease outbreak also in queens [6,17,26]. In comparison, queens forced into contact with symptomatic workers under laboratory conditions (group A) more frequently developed symptoms and had very high viral titers. 

Our research thus demonstrates that forced contact of queens with symptomatic worker bees can lead to an overt infection at least as effectively as the topical application of virus suspension, similar to what is known for contact between workers [12]. Onset of symptoms in the queens of group A was observed on day 5–6 p.i., as previously reported for workers who were subjected to the same infection method [12]. Thus, the symptomatic workers in each laboratory cage must have spread and transmitted the virus to the queen very effectively. Since a honey bee queen is continuously tended to and fed by surrounding worker bees, the likely routs of infection within the laboratory cages are direct contact between individuals where virus is transmitted via the epidermal cytoplasm, or trophallactic feeding. Although transmission via feces is also possible, we consider it less likely in case of the queen [8,12,13]. In contrast to the results obtained in the laboratory cages, the onset of symptoms was considerably delayed in the queens that were kept in mating nucs (group C), with significantly lower virus titers reached after 9 days. Although these queens also were subjected to forced close contact with symptomatic workers inside the shipping cage, they had the chance to refuse being fed by their cage mates and instead receive food from the healthy workers in the surrounding mating nuc. The social immunity like self-removal may play a role that infected bees change their behavior and avoid attending or feeding the queen [31,32] or conversely, queen avoids to be attended by infected retinue.

We therefore suggest that in the context of the colony a transmission of virus through trophallactic food exchange with symptomatic bees may play a more significant role than previously estimated, since infection *per os* has been reported as less effective than other routes of virus transmission [16], but see [18]. The queen is fed a protein rich secretion from the hypopharynx glands of workers. Since the highest titers in bees with an overt infection are found in the brain and neighboring tissues, the secretion of the hypopharynx glands may easily become contaminated with virus particles. Given the differences in infections between the laboratory cages and the mating nuc environment, it appears that symptomatic bees are able to transmit active CBPV particles in amounts high enough to cause infection of the queen. At the same time, the queen may possess behavioral strategies to avoid infection in the colony context which may serve as explanation for the observation that often a visibly asymptomatic queen with a few attending workers is among the last survivors of a colony that collapsed due to CBPV. 

## 3. Experimental Section

### 3.1. Virus Stock and Propagation

Naturally infected trembling and hairless honey bees were sampled from colonies suffering from chronic bee paralysis in an apiary of the Bieneninstitut Kirchhain, Germany. The virus was partially purified according to the COLOSS BEEBOOK chapter on virus research in *Apis mellifera* [33]. Briefly, the heads of 20 sampled bees were crushed individually in 500 µL physiological saline at 4 °C. The supernatant was centrifuged at 3000 × g for 30 min and the virus suspension was collected. For the purpose of propagating CBPV, 500 newly emerged bees from a confirmed virus free colony were inoculated as described before [34]. This colony had been previously tested for the following viruses: CBPV [6], BQCV [35], DWV [36], Sacbrood Virus (SBV) [37], and the three closely related viruses Acute Bee Paralysis Virus (ABPV), Kashmir Bee Virus (KBV), and Israeli Acute Paralysis Virus (IAPV) which were tested in a single assay [38]. Following inoculation, the workers were kept in groups of 50 in cages until they started showing symptoms. The cages were supplied with sugar candy and water and placed in an incubator at 30 °C and 65% RH. Approximately 100 symptomatic workers were collected and virus was extracted from the head of each individual as explained above. To confirm that each suspension was free of other viruses, total RNA was purified using NucleoMag 96 RNA kit. For each individual bee, quantitative RT-PCR was performed for all viruses mentioned above. Virus suspensions yielding positive results for other viruses than CBPV were discarded. To make up the suspension used in the infection experiment, all individual suspensions that were free of other viruses were pooled together, filtered using a cellulose acetate membrane 25 mm syringe filter, and diluted to 5 × 10^8^ copies/µL (for virus quantification see below). 

### 3.2. Experimental Assay

In the first experiment, we tested the susceptibility of queens to CBPV. Using one year old queens we investigated the development of symptoms and mortality after topical application of CBPV solution or abrasion of thoracic hair without virus application. Finally, we determined the virus load of each queen after they succumbed to the infection. 

In the second experiment, using young and unmated queens, we compared the efficiency of direct virus application to infection via contact to symptomatic worker bees. In addition, we collected data on the effect of the queen’s environment and food supply on the course of infection. 

#### 3.2.1. Infection Experiment 1: Testing Susceptibility

The queens in this experiment were mated and approximately one year old. They originated from apparently healthy colonies of the Kirchhain institute, which were routinely re-queened in the late summer 2012. The queens themselves did not show obvious disease symptoms. They were divided into three control groups and one infection group. After immobilizing by cooling on ice, the queens received the following treatments:

- Infection group (n = 39): a small area on the thorax was carefully shaved and 4 µL of virus suspension was topically applied. Thus, each queens received approximately 2 × 10^9^ virus copies.
- Control group 1 (n = 10): the queens were immobilized, a small area on the thorax was shaved and 4 µL of distilled water was applied.
- Control group 2 (n = 8): the queens were immobilized, and a small area was shaved, but nothing was applied.
- Control group 3 (n = 10): the queens were immobilized, but no treatment was applied.

The queens were kept with about 30 healthy worker bees in metal cages supplied with sucrose syrup, sugar candy and pollen in an incubator at 28 °C and 65% RH. Symptoms and mortality were recorded several times per day until day 15 post infection (p.i.), at which time all experimentally infected queens had died. Dead queens and workers were collected immediately to be frozen at −80 °C until RNA extraction. On day 15 all remaining live queens were also collected and frozen at −80 °C. 

#### 3.2.2. Infection Experiment 2: Comparing Infection Routes

A total of 90 queens (one day old and unmated) were divided into three groups which received the following infection treatments:

- Group A (n = 29): to simulate a natural infection via feeding and contact, queens were kept in metal cages together with about 30 workers each who all suffered from a natural infection with CBPV. These workers were symptomatic and most likely each contained more than 10^7^ virus copies [6]. To minimise the spread of virus via feces, a piece of paper was placed on the floor of each cage and exchanged daily.
- Group B (n = 32): queens were kept with about 30 healthy bees each in metal cages. Following immobilization on ice, 4 µL of virus suspension (5 × 10^8^ copies/µL) was applied to a small shaved area on the thorax of the queens. After the virus suspension was absorbed, each queen was returned to her cage. All cages were supplied with sucrose syrup, sugar candy and pollen cake.

These two groups of queens were kept together in the same incubator at 28 °C and 65% RH. Symptoms and mortality were recorded daily. After observing symptoms in the first queens on day 6 p.i., all queens from both groups were collected in individual tubes and immediately frozen at −80 °C for virus quantification.

- Group C (n = 29): queens were kept in plastic queen shipping cages [39] together with 10 workers showing symptoms of a natural infection with CBPV. Each cage was placed into separate mating nucs [39] containing approximately 1500 healthy bees. Thus, the queens were in close contact with infected bees, but could be fed by workers outside the queen cage. Symptoms and mortality were recorded daily. After observing symptoms in the first queens on day 9 after caging, all queens were collected in individual tubes and immediately frozen at −80 °C for virus quantification.

### 3.3. Virus Quantification

The head of each queen was placed in a 2 mL tube together with a metal bearing ball and freeze-dried on a Heto Lyopro 6000 for three days at 0.009 hPa and −93 °C. Samples were homogenized on a Geno Grinder 2000 for 30 s at 500 Hz. The homogenized head was used for total RNA extraction using NucleoMag 96 RNA kit (MACHEREY-NAGEL, Düren, Germany) on a Kingfisher Magnetic Extractor following the manufacturer’s guidelines. The extracted RNA was stored at −80 °C until further use. 

A two-step real-time RT-PCR assay was used to quantify CBPV in the samples. First, cDNA was synthesized using High-Capacity cDNA Reverse Transcription Kit (Applied Biosystems, California, USA). Thereafter, the synthesized cDNA was diluted 10 fold in RNase/DNase-free water to use for quantitative PCR amplifications. 

Quantitative PCR amplifications were carried out on a vii7 apparatus (Applied Biosystems) in duplicate for each sample using SYBR^®^ Green DNA binding dye (Applied Biosystems). A final volume of 12 µL for qRT‑PCR was loaded on an optical 384 well PCR plate (ABgene) with a primer concentration of 0.4 µM. RNase-free water was used as template for two negative controls (No Target Control (NTC) and No Reverse Transcriptase (NRT)) [40]. The thermal cycling conditions were 2 min at 50 °C (degradation of carryover DNA by uracil-*N*-glycosylase (UDG)), 10 min at 95 °C, followed by 40 cycles consisting of denaturing at 95 °C for 15 s, annealing/extension at 60 °C for 35 s. Virus loads in each sample were quantified using absolute quantification methods based on standard curves obtained through serial dilutions of known amounts of the amplicons as described before [41]. The reference gene β-Actin was included in the analysis as an internal control [41] (Table 3). All pipetting steps were done on an ePMotion 5070 robot (Eppendorf, Hamburg, Germany).

**Table 3:** Primers used for CBPV and β-Actin RT-PCR.

Target	Primers name	Primer sequence	Product size (bp)	Reference
CBPV	F-CBPV	5'-CGCAAGTACGCCTTGATAAAGAAC	101 bp	[6]
	R-CBPV	5'-ACTACTAGAAACTCGTCGCTTCG		
β-actin	F-β-Actin	5'-TGCCAACACTGTCCTTTCTGGAGGT	96 bp	[41]
	R-β-Actin	5'-TTCATGGTGGATGGTGCTAGGGCAG		

### 3.4. Statistical Analysis

The normality of residuals from estimated CBPV genomic loads in queens were tested using the Kolmogorov-Smirnov test and the Shapiro-Wilk normality test. Since the distribution of the residuals was non-normal, a rank transformation was performed to obtain normal distributed data. The data were analysed by one way analysis of variance (ANOVA). The mean values of different groups were compared using Least Significant Difference (LSD) test and the term significant has been used to indicate differences for which *p* ≤ 0.05. Data handling and visualisation was done by R [42]. 

## 4. Conclusions

The results of this study reveal that CBPV successfully replicates in queens and the disease progresses in much the same way as is known for worker bees [6,17]. Symptoms include trembling of legs, and spread and disjunct wings. In some queens, we also observed a bloated abdomen. The results from the first experiment demonstrate that queens have no special immunity to the virus. As the virus settles on the queens’ thorax, in the standard method applied to workers, it will propagate and infect the queen. Even just shaving the thorax to open the cuticle, without application of virus, can result in already present surface virus entering the queen and causing infection. Such viruses could come from hairs broken off from infected workers. These hairs can float easily in the dense hive.

The results from the transmission experiment suggest that behavioral patterns are in place to protect the queen from infection by symptomatic bees. We cannot decide from this experiment if queens avoid contact with or uptake of food from diseased workers, or if diseased workers try to avoid interaction with the queen. In this experiment, queens were exposed to virus both via the epidermal cytoplasm and trophallactic feeding. Confining queens into cages with exclusively symptomatic bees resulted in infective infection, similar to those queens with virus suspensions topically applied. In contrast, the onset of symptoms was considerably delayed in queens caged with symptomatic bees, but with access to healthy workers on the outside of the cage. These queens were exposed to virus via the epidermal cytoplasm, but they had the chance to be fed by healthy worker bees through the netted cage.

The results of this study suggest that behavioral strategies, such as avoidance of contact between infected worker bees and queen, or even infected workers leaving the hive altogether, play an important role in keeping the queen safe from exposure to CBPV inside a colony.
